# Editorial: The impact of social media, gaming, and smartphone usage on mental health

**DOI:** 10.3389/fpsyt.2024.1367335

**Published:** 2024-01-25

**Authors:** Justin Thomas, Fahad Al-Beyahi, Carl Gaspar

**Affiliations:** ^1^ Sync, Digital Wellbeing Program, King Abdulaziz Centre for World Culture (Ithra), Dhahran, Saudi Arabia; ^2^ Department of Psychology, College of Natural and Health Sciences, Zayed University, Abu Dhabi, United Arab Emirates

**Keywords:** digital wellbeing, cyberpsychology, addiction, gaming, social media

In 2019, the World Health Organization ratified the inclusion of “gaming disorder” in its official diagnostic system, the 11th revision of the International Classification of Disease (1). That year also saw a raft of new legislation proposed to the US Senate, such as the SMART, Detour, and Filter Bubble Transparency Acts. These proposed laws aimed to regulate social media platforms in the interests of public mental health. Around the same time, the Chinese government enacted laws targeting video game play. One of the initiatives was a curfew prohibiting minors from playing video games between 10 pm and 8 am, with the responsibility for implementation primarily placed on the gaming industry (2). All these legislative and nosological moves reflect a growing global concern about the potential adverse impacts of digital technology on our physical, mental and social health.

Research, however, has not kept pace with our concerns or, indeed, with the advent and proliferation of new digital technologies. The paucity of conclusive evidence concerning the psychological harms (or safety) of digital technologies has frequently led to premature conclusions, with tentative speculation often distorted and broadly amplified by media hyperbole. One such notion is that screen time (time spent on digital technology), especially social media, is unequivocally associated with, and perhaps even causative of, poorer psychological well-being. While several studies report such associations (3, 4), others don’t, and some even find positive links in specific contexts (5, 6). Further research, with greater nuance and methodological sophistication, is required.

A significant challenge for empirical research exploring the mental health implications of digital technologies (tech) is that these electronic tools, services, and platforms evolve rapidly. Progress in the tech world is frequently characterized by radical - disruptive - impacts. Conversely, methodologically robust research moves much slower, typically inching forward incrementally. Furthermore, digital technologies, such as the internet, are global in their reach. At the same time, much of the research to date has focused on populations within individual countries, typically the high-income nations of the global north. However, patterns of usage and associations observed in the global north may not be applicable across cultures or other world regions. For instance, rates of gaming disorder symptomatology vary significantly by nation and world region (7), as do rates of problematic social media use.

Cognizant of these current challenges, this Research Topic explores the use of digital technology and its potential impact on mental health from diverse perspectives across numerous world regions. Several of the articles in this Research Topic explore the socio-demographic correlates of problematic technology use among citizens of lower-and middle-income nations. Al-Mamun et al., for example, examine problematic technology use among university students in Bangladesh, while Thomas et al. perform a similar epidemiological exploration across 30 nations with broad representation from countries outside of Europe and North America.

Beyond the multinational focus, the Research Topic also focuses on relatively neglected populations. For example, Guo et al. explore internet use and depression among older adults. Considering current demographic transitions (e.g., increased longevity and falling birth rates) and global population ageing, this is a knowledge gap that requires addressing,

Several of the studies in this Research Topic also aim to explore the impact of the COVID-19 pandemic on technology use. An obvious consequence of the pandemic is that more people than ever before are now working remotely, with a greater deal of their working lives being spent online via digital technology (8). A previous review (9) exploring the mental and physical health effects of remote working reported a broad array of associated problems, including stress, depression, fatigue and reduced quality of life. Exploring technology use during the COVID-19 pandemic offers us potential insights into the mental health implications of our increasingly digitized lifestyles.

We are entangled in a web of digital technologies, from occupation functioning to recreational pursuits. This Research Topic contributes to a broad and evolving evidence base concerning the links between technology use and our mental health. We hope this Research Topic encourages further research at this critical human-computer interface.

## Author contributions

JT: Writing – original draft, Writing – review & editing. FA-B: Writing – original draft, Writing – review & editing. CG: Writing – original draft, Writing – review & editing.
